# Current research landscape and future prospects of in silico modeling approaches for atopic dermatitis

**DOI:** 10.1016/j.xjidi.2026.100513

**Published:** 2026-07-21

**Authors:** Hiu Lam Athena Wu, Pierre Le Floch, Ariane Duverdier, Alan D. Irvine, Sandrine Dubrac, Reiko J. Tanaka

**Affiliations:** 1 Department of Bioengineering, Imperial College London, London, United Kingdom; 2 Department of Computing, Imperial College London, London, United Kingdom; 3 UKRI Centre for Doctoral Training in Artificial Intelligence for Healthcare, Imperial College London, London, United Kingdom; 4 Clinical Medicine, Trinity College Dublin, Dublin, Ireland; 5 Department of Dermatology, Venereology and Allergology, Medical University of Innsbruck, Innsbruck, Austria

**Keywords:** 3R, Atopic dermatitis, In silico models, Machine learning

## Abstract

Atopic dermatitis (AD) is a chronic, multifactorial inflammatory skin disease with a complex, heterogeneous pathogenesis. Understanding its mechanisms, stratifying patients into biologically relevant endotypes, and predicting treatment responses remain challenging if we use empirical approaches alone. In silico approaches, including mathematical modeling, statistical and machine learning methods, enable the dissection of molecular and cellular interactions, the identification of key clinical and biological drivers, and the extraction of meaningful insights from high-dimensional, noisy datasets, while preserving a systems-level perspective. This review summarizes recent advancements in in silico approaches for AD and outlines strategies to enhance their translational and clinical utility in AD research.

## Introduction

Atopic dermatitis (AD) is 1 of the most common chronic inflammatory skin conditions worldwide, with a prevalence ranging from 2 to 36% in children and from 1 to 18% in adults with notable variation across world regions and a higher prevalence reported in urbanized compared to rural settings, particularly in children (Bylund et al, 2020; Kowalska-Olędzka et al, 2019; Shin et al, 2023). AD patients share clinical characteristics such as relapsing pruritic eczematous skin lesions, skin microbiome dysbiosis (Kong et al, 2012; Nakatsuji et al, 2017), and dysregulated immune responses. However, multiple endotypes of AD highlight the high heterogeneity of microbial signatures and immunophenotypes across patients (Tay et al, 2021; Tokura and Hayano, 2022). Moreover, AD is a multifactorial disease that involves a complex interplay between genetic factors (eg, *FLG* pathogenic variants) (Stefanovic and Irvine, 2024), immune abnormalities (Leung and Guttman-Yassky, 2014), and environmental triggers such as allergens and microbes (Stefanovic et al, 2021). Our current understanding of the pathomechanisms driving AD and responsible for the multiple endotypes is still limited, hindering the systematic design of personalized therapy.

Various ex vivo and in vitro human AD skin models, such as human skin explants, human epidermal equivalents, and human skin equivalents, have been developed to advance our understanding of the disease (Bolle et al, 2020; Burian et al, 2021; Leman et al, 2019; Liu et al, 2020a; Rikken et al, 2023). In recent years, 3-dimensional (3D) skin models have been developed to more accurately recapitulate the human AD skin environment, leveraging specific gene variants, tailored culture media, and defined cytokine cocktails to mimic key pathological and immunological features (Danso et al, 2014; Kamsteeg et al, 2011; Kim et al, 2022). A recent consensus paper critically reviewed human AD skin models and provided information on their advantages, limitations, and opportunities for improvement (Peters et al, 2026). Nonetheless, deciphering the key pathogenetic mechanisms of AD and accurately modeling distinct endotypes remain challenging due to the intricate interplay between multiple cell types, signaling pathways, and molecular processes that occur simultaneously in experimental systems. The use and application of mathematical (or in silico) models and computational approaches have therefore gained increasing interest in the field of AD research.

In silico models of AD are developed by integrating knowledge and empirical data on disease pathogenesis obtained from literature, experimental, and clinical studies. The 2 best**-**established in silico models are mechanism-based models that focus on understanding causality in disease pathogenesis and data-driven models that explore associations between observed data. A review on human and computational models of AD published in 2019 (Eyerich et al, 2019) included mechanistic models that mainly focused on understanding the system-level and gene regulatory mechanisms during disease onset and progression (Christodoulides et al, 2017; Domínguez-Hüttinger et al, 2017; Polak et al, 2017; Subramanian et al, 2018). Pharmacological models based on clinical trial data have also been developed to elucidate the effects of individual variability on treatment response (Kovalenko et al, 2016; Saito et al, 2017). However, the application of data-driven models based on statistical and machine learning (ML) methods was still limited at the time (Kiiski et al, 2015; Thijs et al, 2017).

Advancements in experimental techniques and sequencing technologies over the past years have seen a step change in the volume of data that are available for analysis. As a result, rapid progress has been achieved in in silico and artificial intelligence (AI)-related research on AD, including research on host-microbiome interactions, computer simulations to assist systematic design of treatment strategies, and applications of statistical and ML methods to analyze biological and imaging data. These approaches have enabled image-based disease diagnosis, biomarker discovery, identification of clinical predictors, and prediction of disease severity, as well as inter-individual variability in treatment responses, thereby providing a basis for future precision medicine strategies in AD research. Building on a previous publication (Eyerich et al, 2019), this review summarizes recent advancements in the research landscape over the past 7 years (Table 1) and proposes future directions to further enhance the predictive power and clinical utility of in silico models in AD research.

**Table 1:** Summary of Research Work on In Silico Modeling Approaches for AD

Model Type	Reviewed in (Eyerich et al, 2019)	New Publications up to 2025
Mechanistic mathematical models	(Christodoulides et al, 2017; Domínguez-Hüttinger et al, 2017)	(Lee et al, 2024; Pandey et al, 2023)
Petri net	(Polak et al, 2017)	—
Pathway-based models	(Subramanian et al, 2018)	—
QSP	—	(Go et al, 2024; Miyano et al, 2022a, 2022b)
Population PK/PD	(Kovalenko et al, 2016)	—
	(Saito et al, 2017)	
Image-based diagnosis	—	(Hameed et al, 2020; Rasheed et al, 2022; Tanaka et al, 2021; Yanagisawa et al, 2023)
Disease monitoring and severity assessment	—	(Bang et al, 2021; Maulana et al, 2023; Medela et al, 2022; Moreau et al, 2018)
Prediction and modelling of AD dynamics	Kiiski et al (2015)	(Huang et al, 2021; [Bibr bib32][Bibr bib29], 2020; Nielsen et al, 2025; Nymand et al, 2024)
Biomarker discovery and endotype identification	Thijs et al (2017)	(Ma et al, 2025; Maintz et al, 2023; Möbus et al, 2022; Thijs et al, 2017)
Treatment response prediction and drug discovery	—	(Hurault et al, 2024^1^; Peng et al, 2025; Silverberg et al, 2021; Wu et al, 2022; Yamanaka et al, 2023)

Abbreviations: AD, atopic dermatitis; PD, Pharmacodynamics; PK, Population Pharmacokinetics; QSP, quantitative systems pharmacology.

1 Hurault G, Stalder JF, Aroman MS, Tanaka RJ. Data-driven personalised recommendations for eczema treatment using a Bayesian model of severity dynamics. medRxiv 2024.

## Results

### Mechanism-based models

Mechanism-based models can help overcome some of the limitations of ex vivo and in vitro models, including a possibility to test hypotheses under challenging biological conditions. Furthermore, mechanism-based models can be tailored to focus on specific interactions of interest by filtering out biological “noise” and complex crosstalk while preserving a systems-level perspective. This approach not only enables the identification of critical knowledge gaps but also informs the rational design of experimental studies and clinical trials, enhancing the efficiency and translational relevance of AD research.

To date, 5 main categories of mechanism-based models have been developed for AD, namely mechanistic, Petri Net, pathway, quantitative systems pharmacology (QSP), and population pharmacokinetics/pharmacodynamics models. Below, we will focus on reviewing areas with new publications over the past 7 years.

#### Mechanistic models

Mechanistic modeling aims to understand and elucidate mechanisms that underpin disease pathogenesis and treatment response. Depending on the research focus and mechanism of interest, these models can be constructed and explored at different biological scales, ranging from community-level models that study microbial interactions between species resident on the skin to cellular and subcellularlevels that depict immunological responses and cell signaling. Recent mechanistic modeling efforts in AD have expanded beyond studying disease onset and progression (Domínguez-Hüttinger et al, 2017) to also investigate the roles of immune (Pandey et al, 2023) and host–microbiome responses (Lee et al, 2024).

Pandey et al, 2023 studied the mechanisms driving the transition between T helper (Th) 1- and Th2-dominant endotypes in AD. Building on previously reported findings on enhanced Th1 inflammation in chronic AD versus predominant Th2 inflammation in acute AD, they modeled cellular interactions driven by 2 cytokines, namely, IFN-γ (Th1) and IL-4 (Th2), and their respective effects on the dynamics of Th1/Th2 cells, keratinocytes, and dendritic cells in a set of 4 ordinary differential equations (ODEs). They reported that the transition from Th2-dominant to Th1-dominant AD endotypes can occur either through a gradual or abrupt reduction in Th2 cytokine levels or, alternatively, through an increase in Th1 cytokine levels. In line with this, dupilumab, an IL-4Rα antagonist used for treating AD, can cause a paradoxical, drug-induced reaction resulting in de novo or flared psoriasis/psoriasiform dermatitis. This reaction typically presents as plaques within 6 to 12 months, especially on the face or neck, due to an immunological shift toward Th1/Th17 predominance (Maronese et al, 2024). Findings of this study may pose a question regarding the effects of anti-Th2 therapeutic approaches for treating AD at the molecular level, as well as their consequences on a Th1-dominant disease endotype. Alternatively, deep phenotyping of patients treated with dupilumab might challenge the results of this study.

Lee et al (2024) developed a mechanistic model that describes the dynamic relationship between the pathogen *Staphylococcus* (*S*) *aureus*, the skin commensal *S epidermidis*, and the epidermal barrier integrity through a set of ODEs to explore the mechanisms driving pathogen-induced skin damage in AD lesions. Their in silico model analysis suggests that epidermal barrier recovery is more likely to be achieved when temporary *S. aureus* killing is combined with enhanced growth attenuation of both *S aureus* and *S epidermidis* in treatments. These results highlight the relevance of such models to further study the contribution of dysbiosis to skin damage and disease pathogenesis. Wet-lab skin research can build on these results to propose innovative experiments based on 3D human organotypic cultures or similar skin equivalent modeling systems to decipher the cellular and molecular basis of the interactions among the 2 staphylococcal species and the epidermal barrier in various contexts (eg, primary cells from patients, treatment with cytokines, and genetically engineered cell lines).

#### QSP models

QSP modeling is a computational approach that aims to mathematically capture the dynamics between therapeutic drugs and disease pathophysiology. To date, **3** QSP models have been published specifically for AD.

The 2 QSP models by Miyano et al (2022a, 2022b) were developed by integrating knowledge on the molecular and cellular interactions described in AD with the meta-analysis data from clinical trials (with clinical efficacy normalized for EASI [Eczema Area and Severity Index]). The goal of this approach was to investigate the mechanisms underlying inter-individual variability in therapeutic responses in AD and to guide the development of novel immune- and *S. aureus*-targeted therapies.

Miyano et al (2022a)'s initial QSP model successfully recapitulated the clinical efficacies observed in clinical trials on 9 biologic drugs for AD (dupilumab, lebrikizumab, tralokinumab, secukinumab, fezakinumab, nemolizumab, tezepelumab, GBR 830, and recombinant IFN-γ) by describing system-level influences between pathogen load, epidermal barrier impairment, cytokines, Th cells, and drug effects on AD. The analysis using the QSP model suggested that the baseline level of IL-13 in the skin can serve as a biomarker to predict the response to dupilumab in AD patients and that the simultaneous inhibition of IL-13 and IL-22 could be a novel treatment strategy for poor responders to dupilumab, a proposition that is testable in clinical trials, such as temtokibart in phase 2a study (NCT04922021) (Thaçi et al, 2025).

Their second model captures dynamic interactions between *S aureus*, coagulase-negative staphylococci, IL-4/IL-13, and epidermal barrier (Miyano et al, 2022b). The effects of 3 common clinical treatments (flucloxacillin, dupilumab, and topical application of *Staphylococcus hominis* A9) were explored in silico to understand their contradictory therapeutic efficacies reported in clinical trials. In silico *S aureus*-targeted treatments (including *S aureus*-killing alone, or with simultaneous inhibition of *agr* expression) were found to be effective in patients who respond poorly to dupilumab treatment and exhibited enhanced efficacy when used in conjunction with dupilumab.

On the other hand, Go et al (2024) focused on the application of QSP modeling to support optimization of clinical trial designs for AD drug development. Their QSP model depicts a comprehensive interaction network between skin barrier integrity, skin AD biomarkers such as the presence of pathogenic microbial species and reduced filaggrin levels, immune system components (eg, cytokines, chemokines, regulatory T cells, Langerhans cells, and other dendritic cells), and treatment efficacy (emollients, topical corticosteroids, and the immunomodulator OM-85). After validating that their model could reproduce outcomes from an existing OM-85 clinical trial, they conducted a global sensitivity analysis, which identified alarmins, IL-22, Th2 cytokines, and regulatory cytokines as the key parameters most predictive of treatment response to OM-85. Subsequently, by using this panel of shortlisted biomarkers to stratify virtual patients for trial protocol optimization and selecting only the top 5% predicted responders, they were able to reduce the required sample size for achieving statistical significance by 75%, while simultaneously increasing the average treatment effect by 150% and improving the probability of trial success by 33%. These results demonstrate promising proof of concept and represent an important step toward the transition to model-informed personalized therapies and precision medicine, where in silico modeling approaches can be integrated with experimental and clinical workflows to streamline the drug development process and enhance efficiency in resource utilization. However, further experimental and clinical validation will be required before such approaches can be routinely integrated into therapeutic decision-making or clinical trial design.

### Data-driven approaches

In addition to the various types of mechanism-based models described above, the past 7 years have seen a rapid increase in data-driven approaches applied to AD research (Cao et al, 2025; Duverdier et al, 2022; Liu, 2025). Most studies to date have focused on applying established ML methods to key questions in AD research, rather than developing new methods specific to AD. Here, we highlight key areas of AD research where data-driven approaches have been applied, including image-based diagnosis, disease monitoring, prediction and modeling of AD dynamics, biomarker discovery, and treatment response prediction and drug discovery.

#### Image-based diagnosis

Deep learning (DL) image analysis for the diagnosis of skin disease has become a major area of research in recent years (Choy et al, 2023). Accurate detection and differential diagnosis of AD remain challenging in clinical practice, given the disease's heterogeneity and its overlapping clinical appearance with other inflammatory skin (Deleuran and Vestergaard, 2014). With automated evaluation of skin images, ML can assist AD diagnosis by providing an objective and standardized distinction of AD from other dermatoses. Convolutional neural networks (CNNs) trained on digital camera images (Hameed et al, 2020; Liu et al, 2020b; Pangti et al, 2021; Sarı and Keser, 2025; Shanthi et al, 2020; Sharma et al, 2020; Tanaka et al, 2021; Tariq et al, 2025; Yanagisawa et al, 2023; Zhu et al, 2021), ultrasound images (Czajkowska et al, 2021), or advanced imaging modalities such as multiphoton tomography (Guimarães et al, 2020) and optoacoustic mesoscopy (Park et al, 2021) have demonstrated high performance in distinguishing AD from other inflammatory or non-inflammatory dermatoses as well as from healthy skin, including in multiclass eczema subtype classification (Rasheed et al, 2022). These AD diagnosis models flag suspected AD cases for further specialist review. Such tools may facilitate early detection and remote screening, particularly in resource-limited or primary-care settings. However, most of these models have been developed and validated on small datasets, and their generalizability to diverse patient populations and clinical settings remains to be demonstrated.

#### Disease monitoring

ML is increasingly applied to AD monitoring by providing an objective assessment of disease severity and associated symptoms such as itch and sleep disturbance (Huang et al, 2024a). Classical clinical scoring of AD severity based on visual examinations is often affected by inter- and intra-rater subjectivity (Hurault et al, 2022a), whereas data-driven models can offer standardized, unbiased, and automated evaluation. In line with this, DL models have been used to estimate AD severity from digital camera images (Attar et al, 2023; Bang et al, 2021; Junayed et al, 2020; Maulana et al, 2023; Medela et al, 2022; Okata-Karigane et al, 2025; Pan et al, 2020), ultrasound images (Czajkowska et al, 2025), and optoacoustic imaging (Park et al, 2021). These models can capture subtle structural and textural changes in the skin that may not be visible to the naked eye, enabling reproducible scoring of AD severity. Nevertheless, performance may vary across different imaging devices, lighting conditions, and skin tones, and external validation in independent clinical cohorts remains limited. Beyond imaging, wearable sensors coupled with ML analysis have been employed to monitor nocturnal scratching (Au et al, 2023; Ji et al, 2023; Moreau et al, 2018) and sleep disturbance (Mahadevan et al, 2021), allowing objective and continuous tracking of pruritus and sleep quality, which could support disease severity assessment and future treatment decision-making. Despite promising results, the real-world application of such tools is still constrained by patient adherence, device standardization, and integration into clinical workflows.

#### Prediction and modeling of AD dynamics

Beyond diagnosis and monitoring, statistical and ML methods are increasingly used to model the temporal dynamics of AD evolution and to predict disease course at both individual and population levels. Short-term forecasting models, such as Bayesian state-space frameworks (Hurault et al, 2022b, 2022c, 2020) and mixedeffects autoregressive models (Hurault et al, 2021) have been developed to predict future changes in disease severity over days to weeks. For longer-term predictions, boosted decision trees have been applied to cohort data to identify patient characteristics that are most predictive of the following year’s AD severity category (mild, moderate, or severe) and the number of flares (Nielsen et al, 2025). Other studies have used statistical and ML models to address different aspects of risk for the development or progression of AD. For example, birth-cohort data analyses have applied several ML methods, including random forest, to identify children with higher risks of developing AD based on prenatal and early life environmental exposures (Huang et al, 2021). Moreover, nationwide clinical datasets have been used to model pediatric AD prevalence (Landau et al, 2024). Collectively, these models represent an important proof of concept, though further work is needed to establish whether their predictive performance holds when applied to the less structured and more sparsely sampled data typical of routine clinical settings.

Furthermore, cross-sectional deep phenotyping studies have used gradient boosting and multinomial logistic regression to identify the clinical and demographic factors associated with a higher probability of severe AD (Maintz et al, 2021). Similarly, patient-specific mixed-effects models and Bayesian state space models have been used to model AD symptom dynamics and to evaluate whether serum biomarkers or environmental exposures improve short**-**term predictive performance (Hurault et al, 2022b, 2021), while a neural network approach has been used to relate air pollution and climatic variables to AD severity classes, highlighting the contribution of environmental exposures to symptom variability (Patella et al, 2020). Together, these approaches aim to clarify which factors contribute to day-to-day or week-to-week variation in AD severity, albeit through different analytical frameworks. In parallel, long-term phenotyping studies have applied unsupervised ML to stratify AD patients by longitudinal disease course subtypes across their life course. Unsupervised ML has revealed distinct transient and persistent patterns of childhood AD; subsequent analyses have shown that these patterns are associated with early life clinical features, allergic sensitization, genetic risk factors, and immunological endotypes, contributing to disease persistence and heterogeneity (Haider et al, 2023; Lauffer et al, 2021; Mulick et al, 2021; Suaini et al, 2021). For adult AD, unsupervised clustering has identified severity-based phenotypes associated with symptom burden and comorbidities (Nymand et al, 2024). Furthermore, graph-based ML has been used to group AD patients using clinical records, with known gene–pathway information used to suggest mechanistic interpretations for each subgroup (Frau et al, 2025). These findings represent early but promising steps toward data-driven patient stratification in AD, and replication across independent, diverse cohorts will be essential to confirm the broader biological and clinical relevance of the identified subtypes and phenotypes.

#### Biomarker discovery for AD

ML methods have improved the identification of biological markers relevant to AD disease subtyping and therapy response. In skin, ML analysis of biopsy-based transcriptomic data has revealed immune and barrier-related gene signatures that distinguish AD lesional skin from healthy controls. Related ML methods have identified different keratinocyte gene-expression patterns in lesional and non-lesional AD (Martínez et al, 2022) and under different AD treatments (Clayton et al, 2021), fibroblast-driven inflammatory pathways that contribute to AD pathogenesis (Ko et al, 2022), cytokines and chemokines from peripheral blood samples for AD diagnosis (Lee et al, 2023), immune and barrier changes in the stratum corneum (Jurakic Toncic et al, 2021), and protein–lipid patterns that distinguish AD from healthy skin using Raman spectroscopy (Dev et al, 2022).

In blood, ML and clustering methods have identified cytokine-based patient subtypes associated with disease severity, aligning with known IL-13–related biomarkers used in targeted therapy (Maintz et al, 2023), transcriptomic endotypes with distinct eosinophilic immune profiles and differential response-associated signatures under dupilumab treatment (Möbus et al, 2022), and longitudinal changes in serum proteomic, miRNA, and microbial biomarkers during antiIL-4Rα therapy, identifying protein signatures associated with clinical response to treatment (Pažur et al, 2024).

Birth-cohort and early-infancy microbiome studies further show that gut microbial patterns contribute to an increased disease risk. For example, reduced beneficial bacterial taxa and deviations from normal age-dependent gut microbiome development in infancy are associated with the development of AD later in life (Zhang et al, 2019). In addition, interpretable ML methods, including SHapley Additive exPlanations, were used to identify key gut microbial features associated with AD (Ma et al, 2025).

Together, these findings indicate that ML methods can integrate molecular, cellular, and microbiome data to define biologically relevant AD subtypes, support patient stratification for targeted therapies, and highlight early-life microbial signatures that may alert on increased eczema risk. A key challenge in this area remains the biological interpretability of ML-derived biomarker signatures, as data-driven methods may identify statistically robust associations that are difficult to reconcile with established disease mechanisms or to prioritize for experimental follow-up.

#### Treatment response prediction and drug discovery

ML methods are increasingly being explored for predicting treatment response, supporting personalized therapy, stratifying likely responders, and guiding treatment strategy in AD. For example, ML models using electronic health records data have identified clinical predictors of nonresponse to dupilumab (Wu et al, 2022). Similarly, analyses of clinical trial data have helped identify early predictors of response to the JAK inhibitor baricitinib (Silverberg et al, 2021), thereby enabling earlier and more targeted treatment decision-making. Bayesian approaches applied to data from daily symptoms diaries can estimate patient-specific treatment effects and generate personalized short-term management recommendations (Hurault et al, 2024^1^). At the molecular level, ML methods using skin gene-expression data and blood-based molecular profiles have identified gene and protein patterns linked to the absence of response to dupilumab (Peng et al, 2025) or to treatment-related changes during IL-4Rα therapy (Pažur et al, 2024). Blood transcriptomic analysis of various AD endotypes confirms the relevance of this approach when assessing stratified trial designs and treatment strategies (Möbus et al, 2022). While these findings are encouraging, most treatment response models have been developed retrospectively from clinical trial datasets, which may not capture the full heterogeneity of patients encountered in routine clinical practice, and prospective validation remains limited.

Emerging ML frameworks are also used in drug discovery, accelerating the identification and design of new therapies for AD. Network-based and DL approaches have been used to identify key therapeutic targets and screen potential multi-target compounds, including a proposed dual TNFα/IL-4 inhibitor (Wang et al, 2022). In parallel, generative DL models have enabled de novo drug design by generating candidate molecules directly from patient gene-expression profiles, producing compounds predicted to reverse AD-specific transcriptional signatures without requiring predefined therapeutic targets (Yamanaka et al, 2023). These computational drug discovery approaches are still in their infancy; experimental validation of the proposed compounds in preclinical and clinical settings will be essential to demonstrate their therapeutic potential.

## Discussion

### Importance of collecting fit-for-purpose data to improve model generalizability

The primary benefit of in silico modeling is its ability to efficiently explore the varieties of combined effects of AD pathogenesis factors, host responses, drug actions, and experimental conditions. Once established, these models enable rapid, resource-efficient testing of numerous biological and therapeutic scenarios that would be impractical or impossible to evaluate experimentally. Computational simulations can guide experimental design by prescreening biological and experimental conditions of interest before undertaking in vivo, ex vivo, or in vitro experiments. This approach significantly reduces resource use, increases the precision of subsequent wet-lab investigations, and accelerates the research cycle. Importantly, integrating in silico modeling with experimental validation supports the 3R principles (Replacement, Reduction, and Refinement) by minimizing animal use and optimizing study protocols.

However, developing generalizable in silico models that represent diverse populations and generate reliable dynamic model predictions critically depends on the availability of fit-for-purpose data. A lack of fit-for-purpose data can lead to generalizability issues, as medical AI models often show reduced performance when applied to external datasets or clinical settings that differ from the development cohort (Yang et al, 2024). For example, ML-based diagnostic models may exhibit bias toward overrepresented patient subgroups (eg, specific age ranges, ethnicities, or disease severities) if trained on datasets lacking diversity, failing to capture the full spectrum of real-world disease presentations (Liopyris et al, 2022). Such models might be overfitted to the training data during model development, resulting in poor model generalizability and discrepancies when deployed clinically (Gallitto et al, 2025). Similarly, mechanistic models may fail to capture clinically relevant microbiome heterogeneity when developed from datasets with insufficient sampling diversity. To ensure in silico models produce reliable and translational results, in silico modeling approaches should be integrated into study design from the outset. Future experimental and clinical studies should be structured around iterative cycles of data collection, model development, and model validation, with data acquisition strategies explicitly designed to meet modeling requirements. This prospective integration, rather than the retrospective application of models to existing data, is essential for developing models with genuine translational utility.

**Figure 1:**
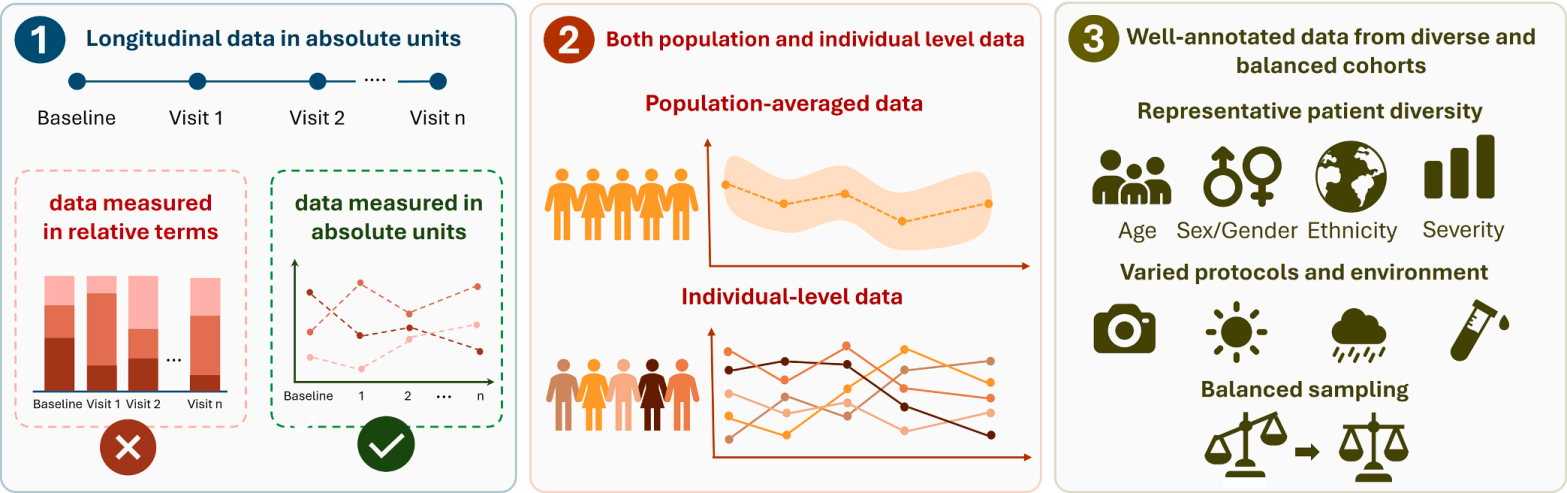
**Three ideal data characteristics for generalizable and clinically-ready in silico models of AD.** Clinically translatable in silico models require longitudinal data in absolute units. Repeated time-course measurements of biochemical mediators, host factors and microbial abundances help capture true biological change over time. Models also require both population-averaged and individual-level data: averaged data help elucidate population-level pathogenic mechanisms, while individual-level data capture inter-patient heterogeneity and reveal individual trajectories. Finally, high-quality, representative, and balanced cohorts with accurate, well-annotated measurements and rich metadata are essential to reduce bias and improve model generalizability. Together, these characteristics form the foundation for developing in silico models with translational utility in AD research and clinical practice. AD, atopic dermatitis.

The development of generalizable and clinically-ready in silico models requires data with 3 ideal characteristics (Figure 1). Firstly, longitudinal data in absolute units are essential. Single time-point measurements are often insufficient to constrain model parameter space. Multiple parameter combinations may produce the same observed data point, making it difficult to uniquely determine system dynamics (Villaverde et al, 2016). Similarly, data measured in relative terms (eg, microbial species concentrations expressed as fractions of total microbial load) may lead to spurious correlations (Gloor et al, 2017). For example, a decrease in relative microbial abundances does not necessarily reflect a true decrease in absolute abundance if the total microbial load of the community changes over time, which is common in microbial cultures and growth phase studies. Time-course data of key host factors, biochemical mediators, and microbial abundances measured in absolute units are therefore critical for adequate model parameterization, enabling reliable interpretation of inferred relationships and predictions generated for disease dynamics and treatment responses.

Secondly, both population-averaged and individual-level data are valuable for model development and validation. While averaged data reduce biological noise and help elucidate population-level pathogenic mechanisms, they may obscure individual trends due to substantial inter-patient variability and the multifactorial nature of AD pathogenesis. Individual-level data are particularly important for understanding disease dynamics, patient monitoring, and predictive modeling, as inter-individual heterogeneity should be factored into consideration for model refinement to accurately characterize disease mechanisms and individual progression trajectories (Lee et al, 2025).

Finally, high-quality input data that are well-annotated and representative of diverse patient populations are essential for the reliability and predictive performance of data-driven and ML approaches. Many current data-driven models for AD are trained on relatively limited datasets that do not always capture the full diversity of patient demographics and disease presentations. Class imbalance or over-representation of specific populations or clinical attributes in training data can introduce diagnostic bias, potentially leading to skewed predictions and poor model generalizability (Arora et al, 2023). To ensure robust real-world performance, the model should ideally be developed using sufficiently large, diverse, and balanced cohort data under varied environmental and technical conditions (eg, imaging protocols). When such balance cannot be achieved during data collection, appropriate bias-mitigation strategies, such as data augmentation, regularization, or post-hoc output correction, should be employed to minimize unintended model bias (Huang et al, 2024b).

Together, these 3 data characteristics, longitudinal absolute measurements, individual-level resolution, and population diversity, form the foundation for developing in silico models with translational utility in AD research and clinical practice.

### Maximizing data value through hybrid modeling approaches

**Figure 2:**
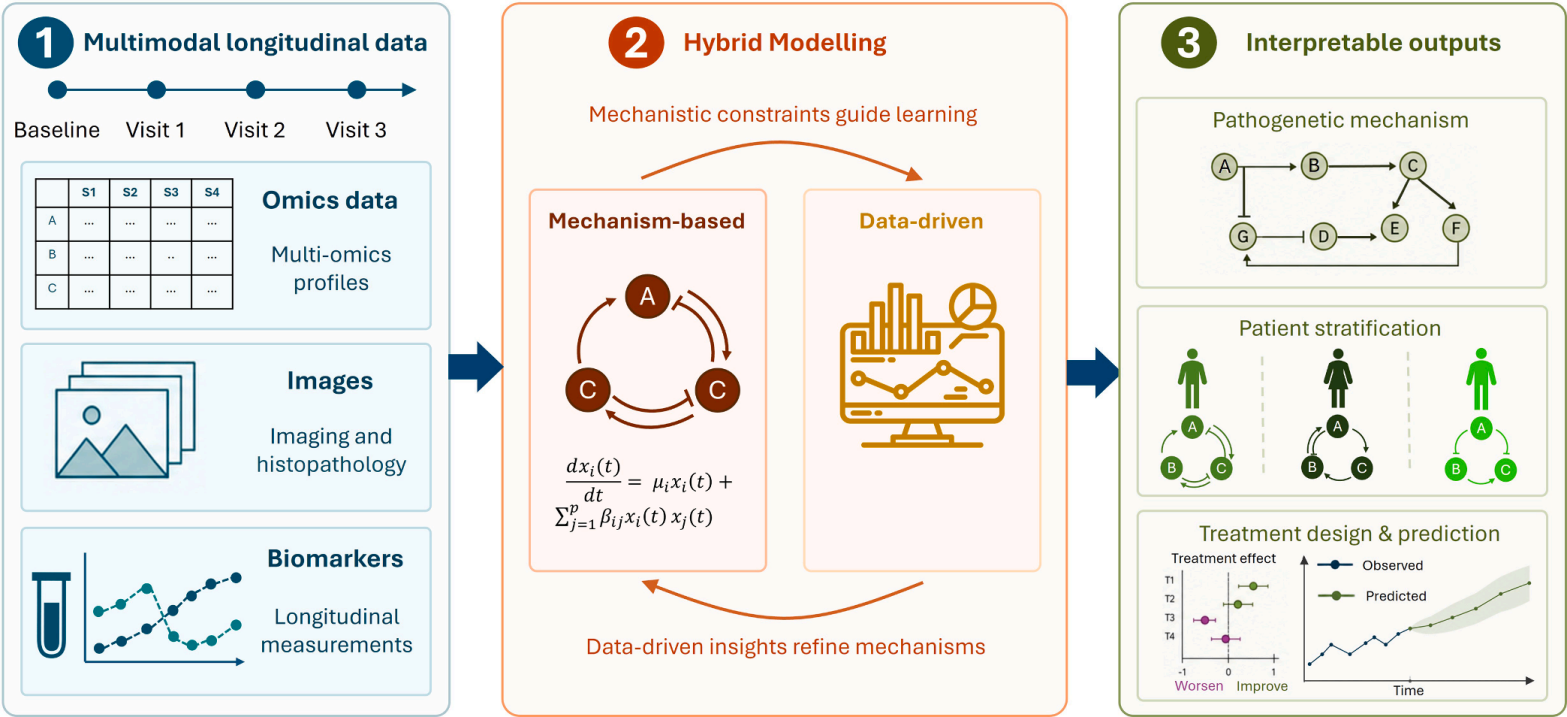
**Hybrid modeling approach combining mechanism-based and data-driven methods.** Multimodal longitudinal data can be integrated to support the development of clinically useful in silico models. In hybrid modeling, mechanistic constraints can guide data-driven learning, while data-driven insights can refine or update mechanistic assumptions. This interaction can improve model interpretability and support the identification of pathogenetic mechanisms, patient stratification, and treatment response prediction, including forecasting of future disease trajectories.

To maximize information extraction and elucidate biologically relevant insights from available data, we advocate for hybrid in silico modeling approaches that combine mechanism-based and data-driven approaches (Procopio et al, 2023). Rather than viewing these approaches in isolation, they should be recognized as complementary and synergistic methods that can be deployed in tandem. Data-driven approaches excel at processing large, high-dimensional datasets and accommodate diverse data types, including image data, which cannot be directly interfaced with mechanism-based models without transformation or translation. However, the results from data-driven approaches may not be readily interpretable or explainable in biologically relevant contexts. Conversely, mechanism-based models provide mathematical frameworks to integrate the existing biological knowledge and connect the model findings to mechanistic insights but may struggle with complex, high-dimensional data. A hybrid modeling approach leverages these complementary strengths (Figure 2). Data-driven methods can extract patterns and features from high-dimensional data, while mechanism-based methods constrain the solution space according to biological insights, ensuring interpretability and mechanistic plausibility. Their synergistic integration enables both powerful data processing and biologically meaningful interpretation.

Hybrid approaches can be implemented in several ways. One strategy uses data-driven feature selection (eg, lasso regression, ridge regression, and elastic net) to identify key microbial species and biomarkers of interest before developing mechanistic models that incorporate these selected variables (Jurakic Toncic et al, 2021). This approach offers a simplified mechanistic model and improves the feasibility for the subsequent validation of model results in controlled experiments.

Another strategy combines ML prediction methods or DL image analysis techniques with mechanistic modeling to explore the pathogenetic mechanisms underpinning AD subtypes, patient stratification, and disease monitoring. For example, a mechanistic ML model framework was proposed for the prediction of AD severity score dynamics (Hurault et al, 2022c, 2020), where a Bayesian state-space model was developed based on a mechanistic model of AD pathogenesis (Domínguez-Hüttinger et al, 2017). This approach leverages ML’s predictive power while maintaining mechanistic interpretability.

Looking forward, physics-informed neural networks (PINNs) represent a promising hybrid approach for AD research. PINNs incorporate mechanistic equations as physics constraints during the DL training, ensuring the biological relevance of model parameters and the mechanistic plausibility of model dynamics. Compared to traditional numerical solvers and optimization algorithms, PINNs offer better handling of continuity when estimating derivatives from sparse or noisy data, a common challenge in clinical AD datasets, where measurements are often irregular and subject to measurement error (Karniadakis et al, 2021; Lin et al, 2025).

### Clinical integration of in silico approaches in dermatological practice

**Figure 3:**
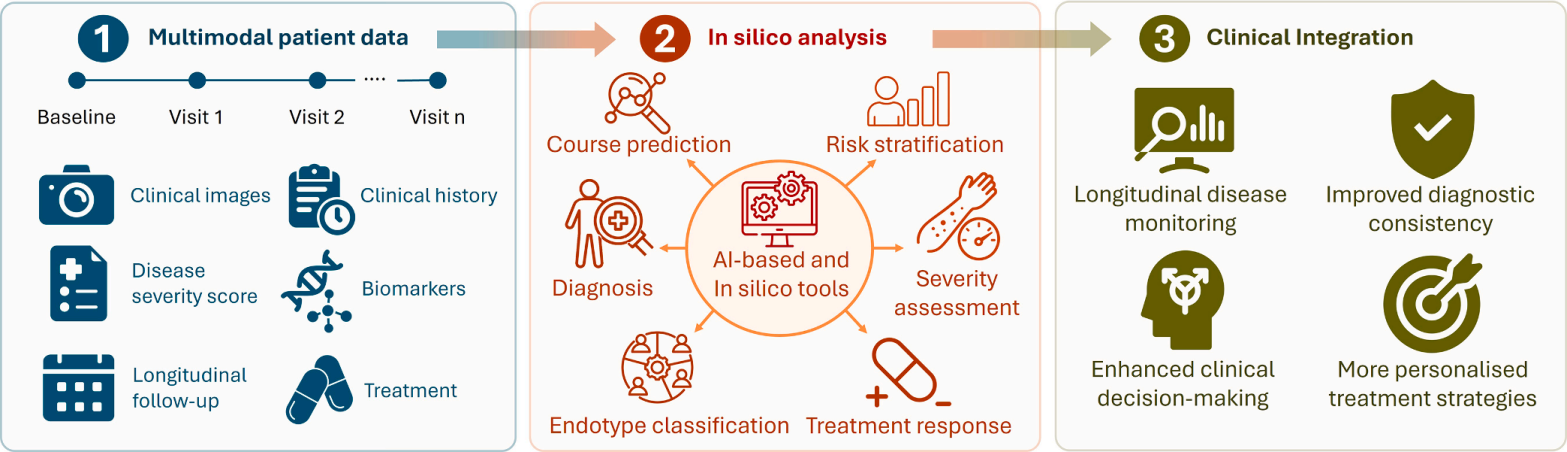
**Clinical integration of in silico approaches in dermatological practice.** Multimodal patient data, including clinical images, severity scores, clinical history, biomarkers, longitudinal follow-up, and treatment information, can inform AI-based and in silico tools for AD. These approaches may support course prediction, diagnosis, endotype classification, risk stratification, severity assessment, and treatment response prediction. Their integration into dermatological workflows could enhance clinical decision-making, improve diagnostic consistency, enable longitudinal disease monitoring, and support more personalized treatment strategies. AD, atopic dermatitis; AI, artificial intelligence.

Beyond their role in advancing computational models of AD, AI-based and in silico tools are likely to be progressively integrated into dermatological workflows in the coming years (Figure 3). These approaches can be informed by multimodal patient data, including clinical images, disease severity scores, clinical histories, biomarkers, longitudinal follow-up data**,** and treatment information. Integrating these complementary data sources may allow in silico tools to capture both the current disease state and changes over time, supporting a more comprehensive assessment of disease activity and patient-specific trajectories.

AI-based and in silico approaches may support several clinically relevant tasks in AD, including diagnosis, disease course prediction, endotype classification, risk stratification, severity assessment, and treatment response prediction. For example, DL models may be used as decision-support tools for diagnosis, while automated image-based scoring systems could improve disease severity assessment by increasing consistency and reducing interobserver variability. Similarly, models trained on combined clinical and molecular datasets may support endotype classification, helping to identify biologically distinct patient subgroups and enabling more precise selection of targeted therapies.

In addition, predictive models may help estimate future disease trajectories, identify patients at risk of severe or refractory disease, and predict treatment responses to therapies such as biologics or JAK inhibitors. These tools may also support longitudinal disease monitoring by integrating repeated measurements across follow-up visits. Overall, the clinical integration of AI-based and in silico approaches has the potential to enhance clinical decision-making, improve diagnostic consistency, enable longitudinal disease monitoring, and support more personalized treatment strategies in AD.

### Concluding remarks

This review summarizes recent advancements in the research landscape over the past 7 years and proposes future directions to further enhance the predictive power and clinical utility of in silico models in AD research.

In silico approaches, ranging from mechanistic models and gene regulatory networks to ML methods, can address diverse research questions in AD research, including a better understanding of disease pathogenesis, characterizing drug mechanisms, supporting disease diagnosis, and predicting disease trajectories. Mechanism-based and data-driven approaches offer complementary strengths. Mechanism-based models can advance our understanding of disease mechanisms through hypothesis-driven testing, supporting the development of novel treatment strategies grounded in mechanistic insight. Data-driven approaches excel at processing and interpreting diverse types of data, including those not directly translatable into mathematical representations, such as clinical images and high-dimensional omics data.

To enhance the translational utility of in silico models in AD research, several advances are needed. First, collection of fit-for-purpose longitudinal data extracted from diverse, well-characterized, and balanced patient cohorts is essential for robust model development, training, and validation. Second, greater integration of mechanism-based modeling and data-driven approaches through hybrid modeling frameworks will leverage the strengths of both paradigms. Ultimately, the full potential of in silico approaches will be realized through systematic integration with experimental and clinical research. Rather than being considered in isolation, computational models should be embedded within iterative cycles of hypothesis generation, in vivo and in vitro experimental validation, therapy design, and clinical evaluation. Such integration will accelerate both our fundamental understanding of AD pathogenesis and the translation of this knowledge into improved patient care.

## Materials and Methods

This manuscript was designed as a narrative review. We searched the PubMed and Scopus databases for mechanism-based and data-driven models, respectively, on 7^th^ November 2025, with no restriction on the starting date. Mechanism-based models were identified using the following string of terms: ("atopic dermatitis" OR eczema OR AD) AND (skin) AND (computational OR "in silico" OR theory OR mathematical) AND (model). Data-driven approaches were identified using the following string of terms: ("atopic dermatitis" OR "atopic eczema" OR eczema) AND ("machine learning" OR "deep learning" OR "artificial intelligence" OR "neural network∗" OR "convolutional neural network∗" OR "CNN" OR "random forest" OR "gradient boosting" OR "boosted decision tree∗" OR "xgboost" OR "support vector machine" OR "SVM" OR "clustering" OR "unsupervised learning" OR "Bayesian model∗" OR "state-space model∗" OR "autoregressive model∗"). All terms had to be present in either the title or the abstract.

The following inclusion and exclusion criteria were applied to the PubMed search results to identify studies that were most relevant to the scope of this review:

- Inclusion
  o Studies that develop or apply in silico modeling approaches for AD in humans. This includes mechanism-based and data-driven methods or applications with a main focus of understanding AD pathogenesis, diagnosis, or generating disease and treatment-associated predictions. through mechanism-based and data-driven methods. Molecular binding and docking studies are excluded.
- Exclusion
  o Studies that are not published in English.
  o Studies that are not original research articles (eg, reviews and opinion papers).
  o Studies that were preprints at the time of the search.
  o Studies of AD in non-human subjects, of the general skin microbiome, or of other skin diseases without clear translation or applicability to AD in humans
  o Studies that focus on molecular structures and interactions (eg, in silico docking) without clear translation or applicability for understanding AD pathogenesis, diagnosis, or generating disease and treatment-associated predictions in humans.

After the initial database searches, titles and abstracts were screened for relevance to in silico modelling approaches in human AD. Full texts were then reviewed to confirm eligibility according to the inclusion and exclusion criteria. Studies were prioritized if they introduced, applied, or evaluated computational models relevant to AD pathogenesis, diagnosis, disease monitoring, prediction of disease dynamics, biomarker discovery, treatment response prediction, or drug discovery. Additional studies not captured by the search strings were included when they were considered directly relevant to the scope of the review, for example, through citation tracking, prior knowledge of key publications in the field, or relevance to specific modelling approaches discussed in the manuscript.

The search results were narrowed down to 4 and 52 studies for mechanism-based and data-driven approaches, respectively, after applying the inclusion and exclusion criteria. Additional studies that were not returned by the search terms were identified based on our knowledge of the field. A total of 11 mechanism-based and 65 data-driven studies were reviewed.

## ORCIDs

Hiu Lam Athena Wu: http://orcid.org/0009-0007-8367-0243

Pierre Le Floch: http://orcid.org/0009-0004-2463-4558

Ariane Duverdier: http://orcid.org/0000-0003-0132-3525

Alan D. Irvine: http://orcid.org/0000-0002-9048-2044

Sandrine Dubrac: http://orcid.org/0000-0002-2936-8488

Reiko J. Tanaka: http://orcid.org/0000-0002-0769-9382

## Conflict of Interest

ADI: AbbVie, Eli Lilly, LEO Pharma, Novartis, Pfizer, Quoin, Regeneron Pharmaceuticals Incorporated, Sanofi, Sitryx, UCB – consultant; AbbVie, Eli Lilly, LEO Pharma, Novartis, Pfizer, Regeneron Pharmaceuticals Incorporated, Sanofi – speaker; AbbVie, Novartis, Sanofi-Regeneron Pharmaceuticals Incorporated – Investigator; Regeneron Pharmaceuticals Incorporated – patent holder. All other authors state no conflict of interest.
